# A Multi-Strain Probiotic Formulation Improves Intestinal Barrier Function by the Modulation of Tight and Adherent Junction Proteins

**DOI:** 10.3390/cells11162617

**Published:** 2022-08-22

**Authors:** Raffaella di Vito, Carmela Conte, Giovanna Traina

**Affiliations:** Department of Pharmaceutical Sciences, University of Perugia, Via Romana, 06126 Perugia, Italy

**Keywords:** intestinal epithelial barrier, probiotics, Serobioma, tight junctions, adherent junctions, intestinal inflammation, Caco-2, TEER

## Abstract

In healthy individuals, tight junction proteins (TJPs) maintain the integrity of the intestinal barrier. Dysbiosis and increased intestinal permeability are observed in several diseases, such as inflammatory bowel disease. Many studies highlight the role of probiotics in preventing intestinal barrier dysfunction. The present study aims to investigate the effects of a commercially available probiotic formulation of *L. rhamnosus* LR 32, *B. lactis* BL 04, and *B. longum* BB 536 (Serobioma, Bromatech s.r.l., Milan, Italy) on TJPs and the integrity of the intestinal epithelial barrier, and the ability of this formulation to prevent lipopolysaccharide-induced, inflammation-associated damage. An in vitro model of the intestinal barrier was developed using a Caco-2 cell monolayer. The mRNA expression levels of the TJ genes were analyzed using real-time PCR. Changes in the amounts of proteins were assessed with Western blotting. The effect of Serobioma on the intestinal epithelial barrier function was assessed using transepithelial electrical resistance (TEER) measurements. The probiotic formulation tested in this study modulates the expression of TJPs and prevents inflammatory damage. Our findings provide new insights into the mechanisms by which probiotics are able to prevent damage to the gut epithelial barrier.

## 1. Introduction

In recent years, scientific research in the fields of gut microbiota and probiotic consumption has risen exponentially, revealing the critical role of microbiota in supporting human health. A large number of commensal bacteria colonize the human gut epithelial layer, providing protection against pathogen invasion. An imbalance of gut flora, on the other hand, may contribute to the onset of intestinal dysbiosis, which is thought to exacerbate a variety of pathological conditions, including inflammatory bowel disease (IBD), diabetes, pancreatitis, non-alcoholic fatty liver disease, and neurological disorders [1,2,3,4,5,6,7,8,9,10]. Moreover, a suboptimal Western lifestyle is associated with dysbiosis and local endothelial dysfunction [11,12].

Notably, evidence is now accumulating in support of the link between dysbiosis, gut barrier dysfunction, and altered intestinal permeability [13,14,15,16]. The gastrointestinal tract serves as a barrier between the internal and external environments and selects nutritive substances from food. Although nutrient absorption is essential for maintaining metabolic homeostasis, a “leaky gut” could create an easy access point for pathogens and pro-inflammatory substances. The gut microbiota, on the other hand, can influence the intestinal barrier integrity, immune response, cardiometabolic functions, and the gut–brain axis [17,18].

The intestinal epithelial layer is the limiting hurdle for nutrient and drug permeation through the gastrointestinal tract. As a result, the integrity of the barrier is considered critical.

Tight junction proteins (TJPs) connect absorptive enterocytes and separate their apical and basolateral membranes. TJ strength regulates the epithelial permeability through the paracellular pathway [19,20], nutrient uptake, waste product clearance [21], intestinal homeostasis [22], and host defense against pathogen invasion [23].

TJ complexes are composed of tetra-spanning membrane proteins (e.g., occludin (OCLN), claudins (CLDNs)), and scaffold proteins such as zonula occludens-1 (ZO-1) and zonula occludens-2 (ZO-2), which bind transmembrane proteins and link them with cytoskeletal actins [24]. However, TJ complexes are dynamic structures, which mutate as a result of stimuli including bacteria and bacterial products [25].

Various studies have shown that disrupted intestinal permeability correlates with a reduced expression and translocation of TJPs. Defects in the TJ architecture of the intestinal barrier could be an etiological factor for various gastrointestinal diseases [21,26].

Several studies reported that probiotics—particularly *Lactobacilli* and *Bifidobacteria* reduce symptoms associated with leaky gut by reducing inflammation, improving the intestinal barrier function, and by promoting the rapid restoration of the gut microbiota [27,28,29,30,31].

Probiotics are defined by the World Health Organization as “*live microorganisms that, when administered in adequate amounts, confer a health benefit on the host*” [32,33]. Probiotics are thought to be a promising strategy for preventing intestinal barrier dysfunction [34,35,36] because they work through a variety of mechanisms, including the competitive inhibition of pathogen adhesion, the production of bioactive metabolites, such as bacteriocins and biosurfactants, the stimulation of digestive enzymes, and the production of short-chain and branched-chain fatty acids [37,38]. *Lactobacilli* and *Bifidobacteria*, for example, have trophic effects on the intestinal mucosa and are increasingly being used to supplement the commensal microbiota in order to maintain a healthy gut microbiota population [39]. Indeed, these strains are normally present in the human intestinal microbiota and are the most widely used in probiotic formulations as supplements for human well-being. Studies have also shown that specific bacterial strains can provide anti-inflammatory, antigenotoxic, and antioxidant activities [40,41,42].

This study aimed to evaluate the protective efficacy of a commercially available multi-strain probiotic formulation, namely Serobioma^®^, in preventing intestinal epithelial barrier dysfunction in an in vitro model of lipopolysaccharide (LPS)-induced inflammation. LPS is a structural component of the outer membrane of Gram-negative bacteria, and it is one of the best-studied immunostimulatory components of bacteria. Generally, LPS concentrations are highest in the intestinal lumen and very low, if not entirely undetectable, in plasma, as LPS does not penetrate through the healthy intestinal epithelium [43]. It has been demonstrated that LPS causes an increase in intestinal TJ permeability [44] by the TLR4-dependent activation of a membrane-associated adaptor protein named focal adhesion kinase (FAK) in Caco-2 cells [45]. Moreover, aspects of great importance are the release of bacteria from the formulation, their viability, and their deposition, so that they are spread along the various tracts of the intestine. On the other hand, although probiotics can adhere to the human intestinal epithelium, various in vitro studies are susceptible to bias in terms of bacterial concentrations, bacterial growth stage, the incubation time, and culture media used [46]. The concentrations used in this study correlate to the number of viable bacteria that reach the gut lumen from the Serobioma formulation.

The permeability of the epithelial barrier was assessed by measuring the resistance of the cellular monolayer. Trans-epithelial electrical resistance (TEER) measurement is a simple, non-invasive technique for evaluating the barrier integrity of the epithelial or endothelial cell layer provided by the TJs strength. We used the human colorectal adenocarcinoma Caco-2 cell line [47] to replicate an intestinal epithelial barrier in an in vitro model because it can form a monolayer of cells that spontaneously differentiate into polarized and columnar enterocytes joined by TJP complexes [48,49]. Changes in the expression of transmembrane TJPs, such as CLDN1, CLDN2, and OCLN, the scaffold proteins ZO-1 and ZO-2, and the adherent junction (AJ) protein E-cadherin (CDH1), were evaluated at the mRNA and protein levels by real-time PCR (RT-PCR) and Western blot, respectively.

## 2. Materials and Methods

### 2.1. Chemicals, Reagents, and Media

DMEM high glucose, fetal bovine serum (FBS), penicillin-streptomycin, trypsin-EDTA, L-glutamine, and Transwell inserts (0.64 mm diameter, 0.4 µm pore size, Costar) were purchased from Euroclone (Milan, Italy). Minimum essential medium non-essential amino acids (MEM-NEAA), SuperSignal^TM^ West Dura Extended Duration Substrate, Pierce^®^ IP lysis buffer, Pierce^TM^ Coomassie Plus Assay Reagent, LPS from *E. coli* 026:B6, TRIzol^®^, High-Capacity Reverse Transcription Kit, and PowerUp^TM^ SYBR^TM^ Green Master Mix were obtained from Thermo Fisher Scientific (Rockford, IL, USA). Skim milk powder was purchased from Microgem (Pozzuoli, Italy). Acrylamide/bis-acrylamide solution 30% (29:1) was obtained from HiMedia Laboratories (Nashik, India). Primary monoclonal antibodies against CDH1 (4A2), CLDN-1 (D5H1D), CLDN-2 (E1H90), OCLN (E6B4R), and HRP-linked anti-mouse and anti-rabbit IgG were obtained from Cell Signaling Technology (Waltham, MA, USA). The primary antibody against β-Tubulin (E-AB-20095) was purchased from Elabscience (Huston, TX, USA).

### 2.2. Cell Cultures

The Caco-2 cell line was established by Dr. Jorgen Fogh (Sloan Kettering Memorial Cancer Center, Rye, NY, USA) in 1974 from a 72-year-old male patient who had previously been treated with 5-fluorouracil and Cytoxan [49]. Caco-2 cells were maintained at 37 °C in humidified atmosphere using DMEM high glucose w/sodium pyruvate, w/L-glutamine supplemented with 10% FBS, 1% MEM-NEAA, 10 U/mL penicillin, and 10 μg/mL streptomycin in 75 cm^2^ flasks. Cells were sub-cultured at a 1:3 ratio when 80% of confluence was achieved. In order to obtain the phenotypic stabilization, Caco-2 cells were cultured for at least two weeks after thawing and sub-cultured at least four times before the experiments [50]. Differentiated cells were used. In brief, Caco-2 cells were seeded at 2 × 10^5^ cells/cm^2^ density and cultured between 18 and 21 days to achieve full monolayer formation and cell polarization. In particular, Transwell inserts and T25 flasks were used for the TEER measurement and RNA/protein extraction, respectively. The culture medium was replaced six hours after seeding [50] and then every two to three days.

### 2.3. Treatment with the Probiotic Formulation

Caco-2 cells were challenged using four different cell/colony-forming unit (CFU) ratios (i.e., 10:1, 1:1, 1:10, and 1:100) for 24 h, in the absence of or in combination with an inflammatory stimulus (i.e., LPS at a 1 μg/mL concentration) added 4 h after probiotic exposure. Serobioma (Bromatech S.r.l., Milan, Italy) is composed of *L. rhamnosus* LR 32, *B. lactis* BL 04, and *B. longum* BB 536.

Prior to each treatment, the Serobioma was suspended in complete medium without antibiotics. Concentrations employed in the present work were selected based on previous research [40].

### 2.4. MTT Assay

Cell viability was determined by the conventional 3-(4,5-dimethylthiazol-2-yl)-2,5-diphenyltetrazolium bromide (MTT) reduction assay, as described by Taticchi et al. [51]. In brief, Caco-2 cells (4 × 10^4^ cells/well) were plated onto 96-well plates until sub-confluence was reached. After treatments, the medium was removed, and the cell monolayers were washed with PBS to eliminate probiotics in suspension. Fresh medium and 10 μL of a 5 mg/mL MTT solution was added to each well. Plates were incubated for 4 h at 37 °C. Finally, the formazan crystals were dissolved in 100 μL DMSO at 37 °C and the absorbance of each well was recorded at 595 nm using a microplate reader. Data were expressed as percentages (%) of reduced MTT, assuming the absorbance of control cells was 100%. Four independent experiments were performed.

### 2.5. TEER Measurements

Cells were treated as previously described. After treatment, cells were washed with sterile PBS and TEER values were registered using a Millicell-ERS Volt-Ohm meter (MilliporeSigma, Burlington, MA, USA) according to the manufacturer’s instructions. In brief, electrodes were equilibrated for two hours in complete medium. The washing step was carried out to remove probiotics adhered to the cell monolayer that could alter the TEER measurements. The measures were repeated three times for each well. The results are obtained from the three independent experiments.

### 2.6. RT-PCR

Cells were treated as described above, and total RNA was isolated using TRIzol Reagent according to the manufacturer’s protocol. Then, 2 μg of RNA was reverse transcribed with a High-Capacity cDNA Reverse Transcription Kit in a 20 μL reaction mixture. The cDNA obtained was diluted at 1:5 and 2 μL of it was amplified and quantified by RT-PCR. The RT-PCR reaction was carried out in a 25 μL mixture containing 400 nM of forward and reverse primers (2 μL), 12.5 μL of ready-to-use PowerUP SYBR Green Master Mix, and RNase and DNase-free water (8.5 μL). As a reference gene, *GAPDH* was used [20]. RT-PCR reactions for each sample and gene were run in triplicate. The PCR conditions were 50 °C for 2 min and 95 °C for 10 min, followed by 40 cycles at 95 °C for 15 s and 60 °C for 1 min. The sequences of the primer pairs were as indicated in Table 1. The mRNA relative expression levels were calculated as 2^−^^ΔΔ^^Ct^.

### 2.7. Western Blot Analysis

After the incubation period, cells were scraped and lysed with ice-cold Pierce IP lysis buffer. Lysates were centrifuged at 12,000× *g* and the supernatant containing the protein extract was collected. The protein amount in each lysate was determined by the Bradford assay. For the Western immunoblotting, 20 μg of total proteins were resolved on SDS-Page gel 8–15% and transferred onto a nitrocellulose membrane (0.2 μm pores). For the immunodetection, nitrocellulose membranes were exposed to the primary antibodies against CLDN-1 (1:500), CLDN-2 (1:500), OCLN (1:4000), and CDH1 (1:1000) at 4 °C overnight and, after, with HRP-conjugated secondary antibodies (1:10,000) (2 h, room temperature). Protein signals were detected using ImageQuant LAS 500 and the ECL kit. The β-tubulin was used as loading control.

### 2.8. Statistical Analysis

The statistical analysis was carried out using SPSS (SPSS Inc., Chicago, IL, USA). The normal distribution was assessed by the Shapiro–Wilk test and a comparison between the treated and untreated samples was then performed by one-way ANOVA followed by Dunnett’s post hoc. Cells co-incubated with Serobioma and LPS were compared with the LPS-treated group. Student’s *t*-test was also used to compare the untreated cells versus the LPS-treated sample at proportions of 10:1 versus 10:1 LPS, 1:1 versus 1:1 LPS, 1:10 versus 1:10 LPS, and 1:100 versus 1:100 LPS. Samples were considered statistically different when the *p*-value was <0.05.

## 3. Results

### 3.1. Cell Viability

**Figure 1:**
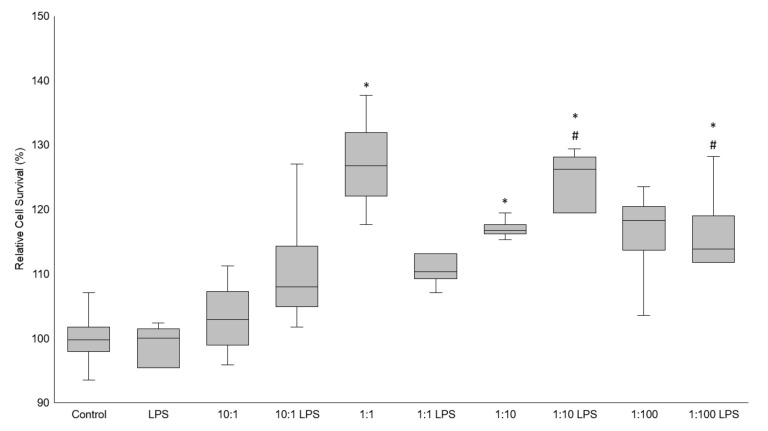
MTT results expressed as the relative cell survival. Caco-2 cells were challenged using four different cell/colony-forming unit (CFU) ratios (i.e., 10:1, 1:1, 1:10, and 1:100) for 24 h, in the absence of or in combination with an inflammatory stimulus (i.e., LPS at a 1 μg/mL concentration) added 4 h after probiotic exposure. Statistical analysis: one-way ANOVA followed by Dunnett’s post hoc. * *p*-value < 0.05 with respect to the untreated control. # *p* < 0.05 with respect to the LPS-treated sample.

Potential cytotoxic effects of Serobioma in Caco-2 cells were determined by measuring the cell viability using the MTT assay after incubation with Serobioma in the presence or in absence of LPS. The results showed that the pre-treatment with Serobioma at all the cell/CFU ratios was not toxic to the Caco-2 cells after 24 h. Quite the opposite, a statistically significant increase in the cell viability was observed at the 1:1 and 1:10 ratios (*p*-value = 0.0004 and *p*-value = 0.0371, respectively). Of note, the pre-treatment of Caco-2 with Serobioma at the ratios of 1:10 and 1:100 was able to induce an increase in the cell viability (*p*-value = 0.0059 and *p*-value = 0.0394, respectively), even in the presence of LPS added 4 h after the probiotic administration (Figure 1).

### 3.2. TEER Measurement

**Figure 2:**
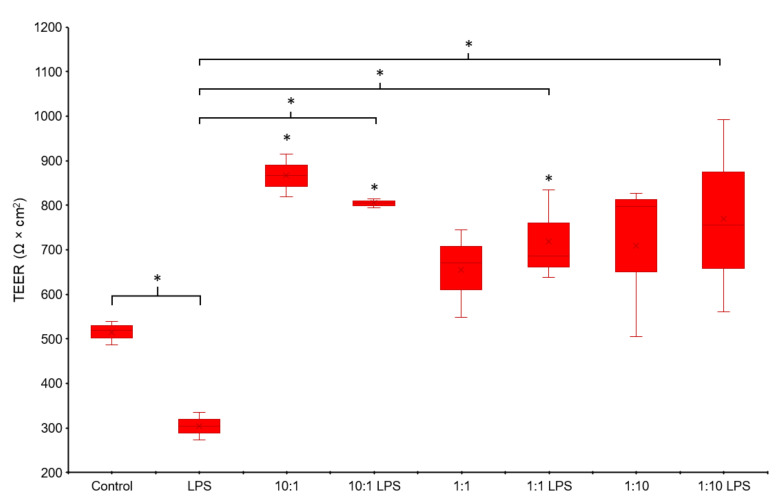
TEER measurement results. Caco-2 cells were challenged using three different cell/colony-forming units (CFU) ratios (i.e., 10:1, 1:1, and 1:10) for 24 h, in the absence of or in combination with an inflammatory stimulus (i.e., LPS at a 1 μg/mL concentration) introduced 4 h after probiotic exposure. Statistical analysis: one-way ANOVA followed by Dunnett’s post hoc. Student’s *t*-test was used to compare control cells against the LPS-treated sample. * *p*-value < 0.05.

Our results show that LPS (1 μg/mL) induced a marked reduction in the TEER values in the Caco-2 cell monolayer after 20 h of exposure (*p*-value = 0.001). Conversely, Serobioma was able to significantly increase the TEER values after the 10:1 cell/CFU ratio exposure (*p*-value = 0.009). Caco-2 cells pretreated with the multi-strain probiotic mixture at the 10:1, 1:1, and 1:10 cell/CFU ratios and then challenged with LPS showed a significantly higher TEER values if compared to the LPS treated cells (*p*-value = 0.002; 0.008; and 0.004, respectively) (Figure 2). Data referring to the 100:1 cell/CFU ratio treatment were not considered, as this concentration led to a rapid acidification of the cell media and affected the TEER measurement (data not shown).

### 3.3. Effect of Serobioma on TJ Gene Expression in Caco-2 Cells

**Figure 3:**
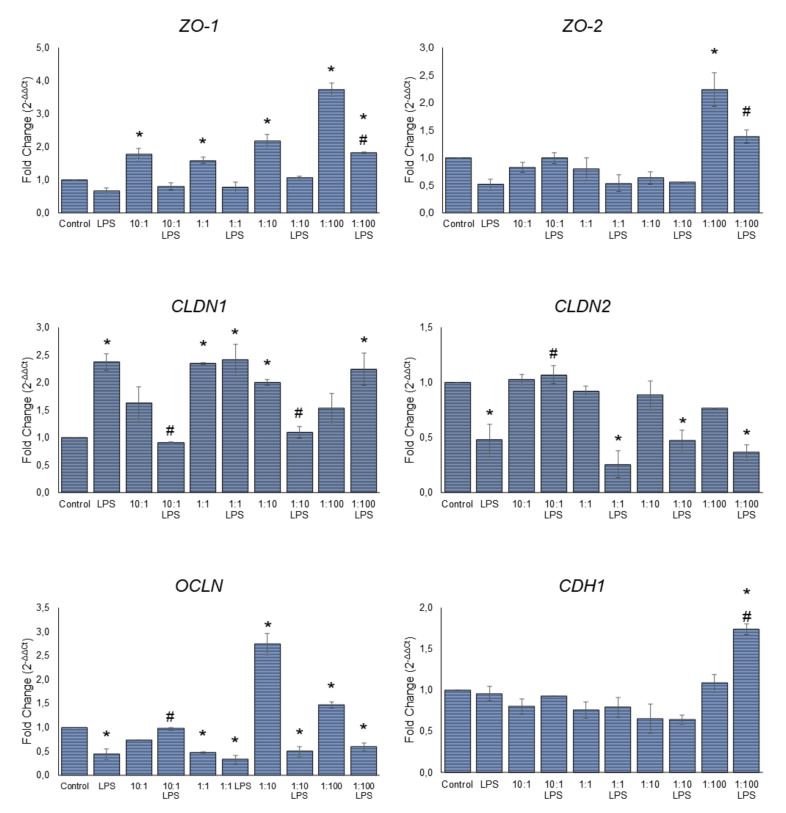
mRNA levels for *ZO-1*, *ZO-2*, *CLDN1*, *CLDN2*, *OCLN*, and *CDH1* genes in the Caco-2 cell monolayer. Caco-2 cells were challenged using four different cell/colony-forming unit (CFU) ratios (i.e., 10:1, 1:1, 1:10, and 1:100) for 24 h, in the absence of or in combination with an inflammatory stimulus (i.e., LPS at 1 μg/mL concentration) introduced 4 h after probiotic exposure. Results are expressed as the fold change (2^−ΔΔCt^) and summarized as the mean ± SEM of three independent experiments. Statistical analysis: one-way ANOVA followed by Dunnett’s post hoc. * *p*-value < 0.05 with respect to the untreated control. # *p*-value < 0.05 with respect to the LPS-treated sample.

To investigate the molecular mechanism underlying the protection exerted by the probiotics, we focused on the expression of six genes encoding TJPs (i.e., *ZO-1*, *ZO-2*, *CLDN1*, *CLDN2*, *OCLN*, and *CDH1*). RT-PCR analysis showed that, compared with the untreated cells, the LPS stimulus induced a significant down-regulation of *CLDN2* (*p*-value = 0.0030) and *OCLN* (*p*-value = 0.0021). For *ZO-1* and *ZO-2*, a statistically insignificant decrease in the gene expression was reported. Conversely, an increase in *CLDN1* mRNA levels was observed (*p*-value = 0.0003). The treatment with Serobioma induced a dose-dependent increase in the expression of *ZO-1* (*p*-value = 0.0044, 0.0344, 0.0001, and <0.0001 for the 10:1, 1;1, 1:10, and 1:100 cell/CFU ratio treatments, respectively). However, the *ZO-2* was up-regulated only by the 1:100 treatment (*p*-value < 0.0001). Meanwhile, compared with LPS alone, the 100:1 Serobioma pre-treatment increased the *ZO-1* and *ZO-2* mRNA levels (*p*-value = 0017 and *p*-value = 0061). As shown in Figure 3, there was a significant up-regulation of *OCLN* at the 1:10 and 1:100 ratios (*p*-value < 0.0001 and *p*-value = 0.0075), and of *CLDN1* at the 1:1 and 1:10 ratios (*p*-value = 0.0003 and *p*-value = 0.0040). The increase in the *CLDN1* gene expression was observed even after the treatment with Serobioma at a 1:1 ratio followed by LPS exposure (*p*-value = 0.0002). Interestingly, a slight reduction in the gene expression of *OCLN* (*p*-value = 0.0027) after the 1:1 cell/CFU Serobioma treatment was also observed. The Figure 3 also shows that the pre-treatment with Serobioma at a 10:1 cell/CFU ratio prevented the LPS-induced changes in the *CLDN1*, *CLDN2*, and *OCLN* mRNA. The 1:10 cell/CFU pre-treatment was able to protect Caco-2 cells against the effect of LPS with respect to the *CLDN1* mRNA levels. Finally, if compared with the untreated control, the 100:1 Serobioma pre-treatment followed by the LPS exposure increased the *CDH1* gene expression (*p*-value = 0.0001).

### 3.4. Protein Expression

**Figure 4:**
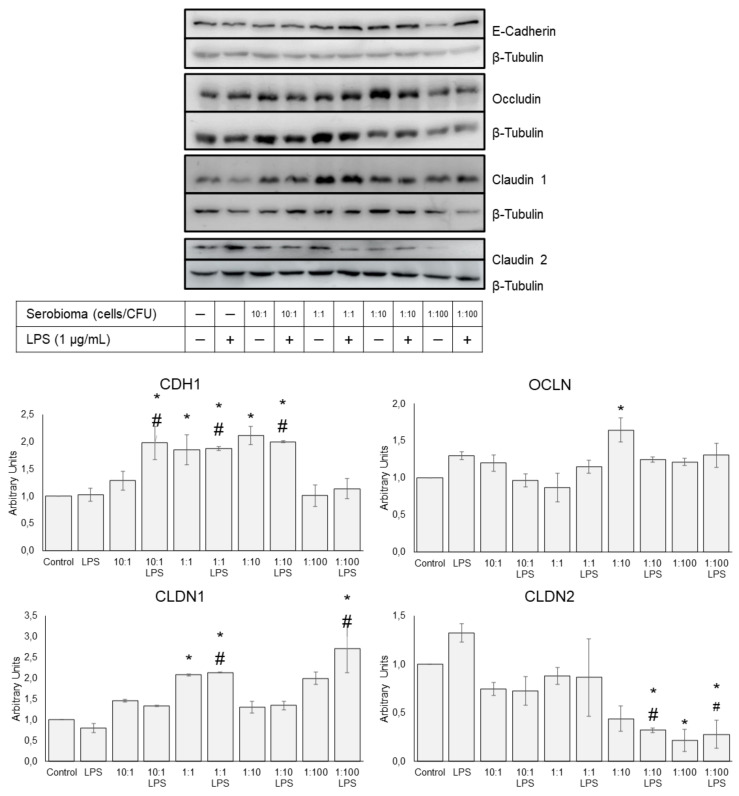
Western blot membranes and results for CADH1, OCLN, CLDN1, and CLDN2 proteins in Caco-2 cells challenged using four different cell/colony-forming unit (CFU) ratios (i.e., 10:1, 1:1, 1:10, and 1:100) for 24 h, in the absence of or in combination with an inflammatory stimulus (i.e., LPS at 1 μg/mL concentration) introduced 4 h after probiotic exposure. The western blot signals were normalized using β-tubulin as the loading control. Results are expressed as mean ± SEM of the protein variation relative to the untreated sample. Statistical analysis: one-way ANOVA followed by Dunnett’s post hoc. * *p*-value < 0.05 with respect to the untreated control. # *p*-value < 0.05 with respect to the LPS-treated sample.

The changes in the TJP amount induced by Serobioma were evaluated by the Western blotting analysis. The protein levels of OCLN, CADH1, CLDN1, and CLDN2 were detected in the Caco-2 cells. Results are summarized in Figure 4. Moreover, uncut and unedited whole membranes are provided in the Supplementary Figure S1. In agreement with the RT-PCR findings, the administration of the probiotic formulation at a 1:1 ratio enhanced the CLDN1 protein levels (*p*-value = 0.022 and 0.015 in presence of LPS), while the CLDN2 protein expression decreased following 1:10 LPS (*p*-value = 0.035), 1:100 (*p*-value = 0.012) and 1:100 (*p*-value = 0.039) treatments. Quite the opposite, LPS decreased the CLDN1 protein levels and increased the CLDN2 levels, in contrast to what was detected using RT-PCR. The CDH1 levels where enhanced after the 1:1 and 1:10 treatments (*p*-value = 0.009 and 0.005), even in presence of LPS (*p*-value = 0.007 and 0.012). Moreover, an increase in the OCLN (*p*-value = 0.006) was observed after Serobioma exposure at the 1:10 ratio.

## 4. Discussion

The aim of this study was to analyze the effect of the commercial formulation Serobioma on intestinal permeability in an in vitro model.

The Caco-2 cell monolayer was exposed to Serobioma for 24 h, in the absence of or in combination with the LPS added 4 h after the probiotic exposure. The 4 h time point for the pretreatment with the probiotic formulation was used, as this is the time required for an efficient metabolism that can impact the bioactivity of probiotics [40,52].

In the present study, the epithelial cell monolayer was challenged with the LPS. The LPS inflammatory damage was confirmed by the reduction of the TEER values in LPS-treated cells compared to the untreated control. Notably, Serobioma was able to prevent the loss of the monolayer integrity at all the concentrations tested (Figure 2). The mechanisms underlying this observation could be multiple and simultaneous, such as the production of bioactive metabolites (e.g., butyrate), the bacterial adhesion that prevents LPS binding, and the modulation of TJPs. For example, it was reported that exopolysaccharide (EPS) produced by lactic acid bacteria (such as *Lactobacilli* and *Bifidobacteria*) could interact with intraluminal water to produce a protective film [53].

Among the strains included in Serobioma formulation, *B. longum* BL536 has been recognized as one of the most effective probiotic strains. It acts mainly through microbiota modulation [54], and it is able to modulate the immune response [55]. Moreover, this strain is capable of stabilizing TJPs through EPS and the production of butyrate as active metabolites [53]. *L. rhamnosus* LR32 exhibited immunomodulatory effects [56,57]. Finally, *B. lactis* BL04 was shown to be useful against dyslipidemia in children [58].

Due to the close relationship between barrier integrity and TJPs, the authors focused their attention on the expression of these proteins.

The administration of the probiotic formulation containing selected species of both *Lactobacillus* and *Bifidobacterium* genera promoted the expressions of *ZO-1*, *CLDN1*, and *OCLN.* These proteins are the most frequently altered during infections and diseases. Indeed, pathogenic microorganisms, such as enteropathogenic and enterohemorrhagic *E. coli*, can alter the barrier properties by affecting OCLN and ZO-1 [59].

A previous study showed that LPS increases the intestinal permeability by affecting OCLN, CLDN1, and ZO-1 and by inducing mitochondrial dysfunction and mitophagy in piglets [60]. In addition, experimental and clinical studies revealed that the downregulation of ZO-1, OCLN, and CLDN1 was associated with increased intestinal permeability in IBD patients [61].

In our study, Serobioma treatment increased CLDN1, while it decreased CLDN2 at both the mRNA and protein levels, suggesting that the probiotic complex proves beneficial and has protective effects, against the LPS inflammatory response. Indeed, CLDN1 is known to form continuous sealing filaments. Conversely, CLDN2 is involved in pore architecture and regulates the paracellular permeability of small ions and molecules. In particular, junctional complexes, in which CLDN2 levels are increased, are characterized by the presence of discontinuous filaments [62]. Likely, in Chron’s disease, the increase in CLDN2 accompanied by the decrease in CLDN1 constitutes the molecular basis of discontinuous filaments, which can lead to the conversion of TJs into leaky junctions [63]. Quite the opposite, LPS decreased the CLDN1 protein levels and increased the CLDN2 levels, in contrast to what was detected using RT-PCR. This apparent discrepancy implies that we observed a reparative response to LPS-associated damage. We also observed a dose-dependent increase in the *ZO-1* expression in the Serobioma-treated Caco-2 cells, suggesting that probiotics play a significant role in improving the integrity of the intestinal epithelial barrier [64].

In the present study, it emerged that CDH1 protein levels were enhanced by Serobioma. However, *CDH1* mRNA levels did not change, suggesting that Serobioma has a time-dependent effect on gene expression or that it activates intracellular pathways that regulate CDH1 turnover. Various studies have reported that CDH1 coordinates the establishment of the apical–basolateral polarity with the formation of adhesion junctions, desmosomes, and TJs [65,66]. CDH1 plays a crucial role in the regulation of actomyosin-dependent tension, TJ positioning, and barrier formation [67]. In addition, the CDH1-dependent cell adhesion system supports enterocyte differentiation and TJ establishment [68,69]. In light of the preceding observations, it is hypothesized that Serobioma also acts during the cell polarization process and aids in the formation of a more robust barrier.

The results presented in this study indicate how the probiotic formulation was able to modulate the expression of TJPs across the epithelial barrier. Overall, the most significant data were recorded when the probiotic bacteria were administered in the rations of 10:1, 1:1, and 1:10 (cells/CFU).

A previous in vitro screening confirmed the anti-inflammatory properties of the Serobioma formulation [40]. Taken together, our results suggest that Serobioma exerts a protective effect against intestinal epithelial barrier dysfunction. In particular, it has a preventive activity against inflammation-associated damage.

We suggest that the molecular mechanisms are attributable to the modulation of pro- and anti-inflammatory cytokine release, the modulation of TJ gene expression, and the activation of inflammatory intracellular pathways.

Our results are corroborated by numerous studies, in which the effectiveness of *Lactobacilli* and *Bifidobacteria* in preserving the intestinal barrier function was evaluated. Beneficial properties of *Bifidobacteria* have been reported in in vivo models of inflammation. For example, feeding with *B. bifidum* promoted the ZO-1 expression in a dextran sodium sulphate colitis mouse model [70]. *B. infantis* has been shown to possess barrier-preserving properties [71]. *B. longum* increased the TEER values and decreased the paracellular permeability of Caco-2 cells stimulated by LPS [72]. *B. longum BB* 536 was able to induce remission in patients with ulcerative colitis [73]. It has been reported that *B. lactis* 420 and *B. lactis* HN019 increased TJ integrity in Caco-2 cells [74]. On the other hand, certain *Lactobacillus* species prevented barrier disruption through the upregulation of TJPs. In addition, *L. acidophilus* and *L. plantarum* increased OCLN expression within in vivo and in vitro models, respectively [34,75]. *L. rhamnosus* CNCM I-3690 partially restored the function of the intestinal barrier and increased the levels of OCLN and CADH1 [76]. The probiotics *E. coli* Nissle 1917, *L. rhamnosus* GG (LGG), and a mixture of *Lactobacilli* and *Bifidobacteria* prevented the increase in intestinal permeability in vivo [77,78,79].

The predictability of the in vitro experimental model is a topic of crucial importance. The in vitro model offers the advantage of evaluating the probiotic effects as such, while minimizing the contribution of environmental factors. However, the environment of the gut is much more complex. The pH of the intestinal lumen may be a determining factor. Indeed, studies reported that acidification elicits the instability of the epithelial TJ complexes in both in vivo and in vitro models [80,81,82]. In our experimental model, the 1:100 cell/CFU ratio could have caused the acidification of the culture medium due to the probiotic metabolism, as ascertained by the medium color shift from red to yellow, which may have had a negative impact on the TEER measurements. Strains of *Lactobacillus* are known to acidify the environment and make it hostile to pathogen proliferation. However, in humans, the mucus layer protects the gut epithelium against excessive acidification. Although it is still difficult to determine whether the in vitro model is predictive of what occurs in vivo, the results of this study provide a rationale for future in vivo and clinical studies.

Finally, there are various forms of commercial probiotic preparations, and their efficacies could differ considerably depending on whether they contain a single strain or multiple strains. Studies have already indicated that the synergistic effects of multi-strain probiotic formulations differ from those of single-strain formulations [83]. In vitro studies have shown that some multi-strain probiotics may show significant better inhibitory effects against enteropathogens and greater benefits when compared to single-strain preparations. Some multi-strain probiotics may reduce the absorption of harmful chemicals due to their ability to absorb heavy metals within their cell walls [84]. Multi-strain probiotics may be able to create a probiotic niche that improves bacterial colonization overall. In particular, in vivo, strains with an optimal pH range of 6–7, typical of the upper intestinal tract, show rapid growth, leading to a decrease in the optimal pH of the most acidophilic bacterial strains [85]. Overall, multi-strain probiotics are more consistent in their actions than single-strain probiotics [86].

For all these reasons, multi-strain probiotic supplementation with Serobioma could be effective method to protect the intestinal barrier function against inflammatory attacks.

## 5. Conclusions

In this study, it was found that the Serobioma formulation, containing a combination of *Lactobacilli* and *Bifidobacteria* strains, is capable of preserving the integrity and functioning of the intestinal barrier from damage caused by the LPS inflammatory stimulus, as well as modulating the expression of genes and proteins belonging to TJs in Caco-2 cell monolayers.

The present study provides additional insight into the mechanisms by which selected probiotic strains prevent intestinal epithelial barrier dysfunction and contribute to sustaining gut health. Our data suggest a broad spectrum of positive effects exerted by the Serobioma formulation, demonstrating a high potential for its therapeutic use in modern medicine. Future studies should aim at analyzing and characterizing other molecular mechanisms and intracellular pathways that mediate probiotic efficacy.

## Figures and Tables

**Table 1:** Primer sequences used for RT-PCR.

Accession Number	Gene Name	Symbol	Product Length	Primer Sequences (F: Forward; R: Reverse)
NM_002046.7	Glyceraldehyde-3-phosphate dehydrogenase	*GAPDH*	120	F: TGACTTCAACAGCGACACCCA R: CACCCTGTTGCTGTAGCCAAA
NM_004360.5	Cadherin 1	*CDH1*	136	F: CTTTGACGCCGAGAGCTACA R: TTTGAATCGGGTGTCGAGGG
NM_002538.4	Occludin	*OCLN*	136	F: ACCAATGCTCTCTCAGCCAG R: AGGCAAAGATGGCAATGCAC
NM_021101.5	Claudin 1	*CLDN1*	170	F: TTTACTCCTATGCCGGCGAC R: GAGGATGCCAACCACCATCA
NM_020384.4	Claudin 2	*CLDN2*	161	F: TATAGCACCCTTCTGGGCCT R: GCTACCGCCACTCTGTCTTT
NM_001301025.3	Tight junction protein 1	*ZO-1*	157	F: GGGACAACAGCATCCTTCCA R: GCAAAAGACCAACCGTCAGG
NM_004817.4	Tight junction protein 2	*ZO-2*	130	F: TTCGTTTGCAGTTCAGCAGC R: CTCAAAAGCCCGGTCATCCT

## Data Availability

All data are included in the manuscript.

## Supplementary Materials

The following supporting information can be downloaded at: https://www.mdpi.com/article/10.3390/cells11162617/s1, Figure S1: uncut and unedited western blot membranes.
