# Supplementary material for: A Multi-Strain Probiotic Formulation Improves Intestinal Barrier Function by the Modulation of Tight and Adherent Junction Proteins
Source: Cells. 2022 Aug 22;11(16):2617. doi: 10.3390/cells11162617 (PMC9406415; doi:10.3390/cells11162617)
Supplement: Supplementary file 1 [file cells-11-02617-s001.zip › cells-1859505-supplementary.pdf]

E-Cadherin (135 kDa) and β-Tubulin (55 kDa)

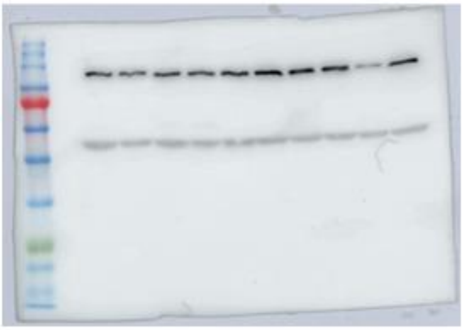

Occludin (65 kDa) and β-Tubulin (55 kDa)

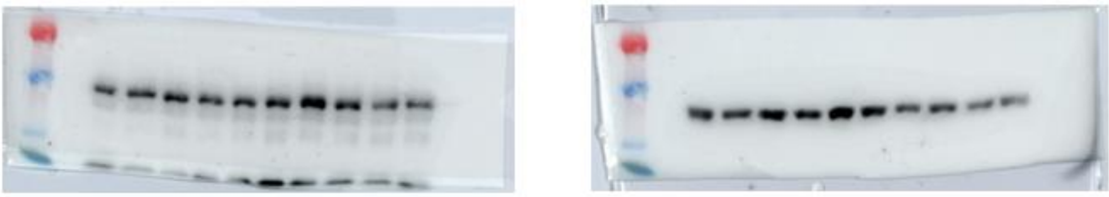

Claudin-1 (20 kDa) and β-Tubulin (55 kDa)

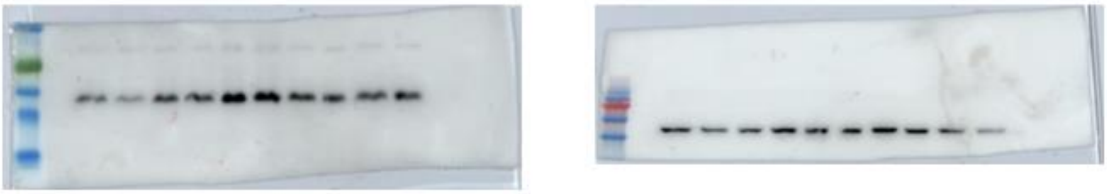

Claudin-2 (20 kDa) and β-Tubulin (55 kDa)

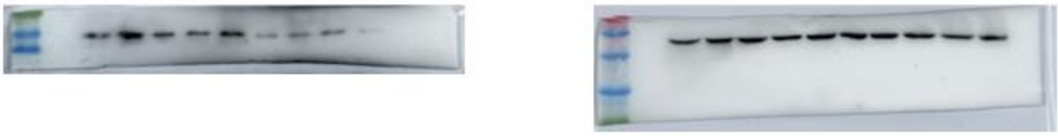

Figure S1. Uncut and unedited western blot membranes.
